# Sexual Violence in Party-Related Social Settings from a Public Health Perspective: A Cross-Sectional Study Among Adults in Poland

**DOI:** 10.3390/healthcare13222963

**Published:** 2025-11-19

**Authors:** Andrzej Silczuk, Olga Płaza, Przemysław Łukasiewicz, Robert Madejek, Agata Olearczyk, Mirosława Palak, Paulina Mularczyk-Tomczewska, Łukasz Czyżewski, Tytus Koweszko

**Affiliations:** 1 Department of Community Psychiatry, Faculty of Life Sciences, Medical University of Warsaw, 02-091 Warsaw, Poland; robert.madejek@wum.edu.pl (R.M.); tytus.koweszko@wum.edu.pl (T.K.); 2 Department of Psychiatry, Faculty of Health Sciences, Medical University of Warsaw, 05-802 Pruszków, Poland; olga.plaza@wum.edu.pl; 3 Addiction Clinic, Dialog Therapy Centre, 02-791 Warsaw, Poland; przemyslaw.lukasiewicz@terapiadialog.pl; 4 Health Innovation Unit, SGH Warsaw School of Economics, 00-641 Warsaw, Poland; aolear@sgh.waw.pl; 5 SexEDPL Foundation, 00-641 Warsaw, Poland; miri@sexed.pl; 6 Department of Public Health, Faculty of Life Sciences, Medical University of Warsaw, 02-091 Warsaw, Poland; paulina.mularczyk-tomczewska@wum.edu.pl; 7 Department of Geriatric Nursing, Faculty of Public Health, Medical University of Warsaw, 02-091 Warsaw, Poland; lukasz.czyzewski@wum.edu.pl

**Keywords:** sexual, violence, party, prevalence, public health, prevention

## Abstract

**Background and Objectives:** Sexual violence constitutes a major public health concern that undermines safety, bodily integrity, and autonomy. This study aimed to assess the prevalence, risk factors, and preventive strategies related to sexual misconduct in party-related social settings in Poland. **Materials and Methods:** A cross-sectional survey was conducted between 18 and 27 March 2025 among a representative sample of 1000 adults using computer-assisted web interviews. The questionnaire covered perceived risk, preventive measures, and personal or witnessed experiences of sexual violence. Data were analyzed using chi-square tests and multivariable logistic regression. **Results:** More than half of respondents reported unwanted touching (53.8%) or persistent harassment (46.9%), and 54.1% had encountered sexual comments. Drug-facilitated assaults were reported by approximately 10% of participants, while 12.3% indicated forced sex. Despite frequent reliance on precautionary measures (e.g., returning home with friends in 64.2% of cases), concerns about sexual victimization were widespread. Multivariable analysis showed that women (aOR 1.91), young adults (aOR 2.80 for those aged 18–24 years), and sexual minorities were disproportionately affected. **Conclusions:** This study highlights that sexual violence in party-related settings is a structural rather than marginal problem in Poland. Women, young adults, and sexual minorities are disproportionately affected. Effective prevention requires multilevel interventions, including bystander programs, venue-level safety policies, and broader public health strategies to reduce tolerance for sexual harassment and violence.

## 1. Introduction

Sexual violence is a severe violation of human rights that undermines safety, bodily integrity, autonomy, and identity. The World Health Organization (WHO) defines it as “any sexual act, attempt to obtain a sexual act, unwanted sexual comments or advances, or acts to traffic, or otherwise directed, against a person’s sexuality using coercion, by any person regardless of their relationship to the victim, in any setting, including but not limited to home and work” [1,2]. Its defining element is the absence of informed, voluntary, and revocable consent. Sexual violence is not an expression of desire but of power and domination.

Sexual violence may be conceptualized within several complementary theoretical frameworks that illuminate its developmental and structural determinants. Feminist theory conceptualizes sexual violence as a manifestation of gendered power asymmetry rather than individual deviance, locating it within broader structures of “rape culture” and normative male entitlement [3]. From a socio-cognitive perspective, social learning theory highlights the mechanisms through which aggression is modeled, reinforced, and normalized in peer environments, a process empirically demonstrated in the context of sexual and partner violence transmission [4]. Multifactorial models conceptualize sexual violence as the result of interacting individual, situational, and cultural determinants, which aligns with public health epistemology and the multi-level explanatory logic applied in this study. A developmental complement is offered by intergenerational transmission theory, which emphasizes that early exposure to coercion or boundary violations increases vulnerability to later perpetration or revictimization [5,6]. Together, these frameworks underscore that sexual violence is not an isolated behavioral incident but a structurally embedded phenomenon shaped by social hierarchies, normative climates, and intergenerational conditioning—features that are particularly visible in nightlife and party-related settings.

Definitions of sexual violence differ across jurisdictions. Some still require evidence of force, while others rely on the principle of consent. Until February 2025, Polish criminal law demanded proof of force, threat, or deception, which restricted prosecution. A legal reform introduced on 13 February 2025 aligned national law with international human rights standards such as the Istanbul Convention by adopting a consent-based definition of rape [7,8,9]. This shift is consistent with broader international human rights obligations. The Beijing Platform for Action (1995) was the first global policy framework to explicitly define violence against women, including sexual violence, as a violation of human rights and to call on states to implement preventive measures [10]. The Istanbul Convention later operationalized these principles within the European legal system by establishing consent-based standards and positive state obligations in the field of prevention and protection.

Drug-facilitated sexual assault (DFSA) is a particularly harmful form, often involving substances such as gamma-hydroxybutyrate (GHB) or benzodiazepines added to drinks without victims’ knowledge. These substances are usually colorless and tasteless, making them difficult to detect. Their effects include disinhibition, memory impairment, and loss of consciousness, often intensified by alcohol [11,12].

Recent systematic reviews indicate increasing sophistication in substance use, with perpetrators utilizing gamma-hydroxybutyrate (GHB), benzodiazepines, and newer designer drugs [11,12,13]. A 2024 Barcelona study analyzed 682 cases of DFSA (among which 85% were women)—the data shows alcohol is still the most common and accounted for 55% of cases, followed by cocaine (28%), MDMA (23%) and GHB (15%) [14], while evolution toward designer benzodiazepines presents new challenges for detection and prosecution [13].

As shown before, epidemiological studies indicate that alcohol itself is the most common substance involved in sexual assaults in nightlife contexts, largely due to heavy episodic drinking rather than covert drugging [14,15,16].

The 2024 review by Lynam and Keatley confirmed that while “date rape drugs” receive significant attention, alcohol-facilitated assaults are still the most common [13], some data suggesting they might account for 55–71% of all DFSA cases [15,17,18].

The scale of the problem is considerable. Globally, one in three women has experienced physical and/or sexual violence during her lifetime [2]. Around 6% of women aged 15–49 report non-partner sexual assault [19]. In the European Union, 17.2% of women have experienced sexual violence, although prevalence varies between countries, from over 40% in Sweden to below 20% in Poland [20,21]. More than half report sexual harassment in public spaces since the age of 15, affecting their sense of safety and freedom of movement [21]. Eurostat data indicate that about 20% of women have experienced sexual violence outside intimate partnerships, including 4% reporting rape [22].

The 2022 study that analyzed the WHO Global Database on Prevalence of Violence Against Women, based on 366 surveys in 161 countries, estimated that up to 492 million ever–partnered women aged 15–49 had experienced intimate partner violence at least once since age 15. Overall, 27% (UI 23–31%) reported lifetime physical or sexual violence, and 13% (UI 10–16%) reported past–year violence. Regional prevalence was highest in Oceania (49%; UI 38–61%) and central sub–Saharan Africa (44%; UI 33–55%), followed by South Asia (35%; UI 26–46%). Country–level estimates ranged from 10% (UI 6–17%) in Georgia and Armenia to 53% (UI 35–70%) in Kiribati [23]. Moreover, sexual minority individuals are subject to substantially higher victimization. According to recent U.S. national data among women, 79.3% of bisexual women and 59.9% of lesbian women experienced contact sexual violence during their lifetimes, compared to 53.3% of heterosexual women. Among men, 59.8% of gay men and 56.4% of bisexual men experienced contact sexual violence, compared to 29.3% of heterosexual men [24,25].

Party environments such as bars, clubs, and festivals concentrate risk factors: crowding, anonymity, sexualized atmospheres, and widespread alcohol use. A systematic review shows that the lifetime prevalence of sexual violence in these settings exceeds 50% in many samples of young patrons [14]. In an English survey, 58% of respondents reported experiencing sexual violence during a night out [26]. Women, especially younger ones, are disproportionately affected, with perpetrators often being peers or acquaintances [14,26].

Recent international research provides important context for understanding party-related social settings of sexual violence. Australian studies report unwanted sexual attention affecting 5–20% (5% males and 20% females) of nightlife attendees [27]. Canadian research documents comparable prevalence rates among university female students frequenting bars and house parties (105 out of 528 cases of sexual assaults happened either in bars or restaurants or at house parties) [28].

Social norms also contribute. In party-related social settings, provocative dancing, physical contact, and sexualized dress are common, sometimes leading to misinterpretation of behavior as consent. Attitudes linking women’s alcohol use with sexual availability further increase risk [26]. Bystander intervention remains infrequent, with studies showing that only about one in five incidents elicit an active response. Prevention, therefore, requires staff training, awareness campaigns such as “Ask for Angela,” and broader alcohol-policy measures including stricter enforcement of serving laws [26,29,30].

The concept of “rape culture” societal norms that trivialize sexual violence is particularly evident in party social environments. UK research on music festivals reveals how sexual violence occurs on a continuum, representing an extension of broader cultural patterns that normalize male sexual aggression [31]. Nightlife environments are characterized by “a cultural atmosphere where instances of unwanted sexual contact such as touching, groping, and other aggressive attempts at coercion, as well as verbal harassment, are normalized.” This represents what researchers term “the cultural scaffolding of rape,” systematic conditions that facilitate sexual violence through the normalization of male sexual aggression and the use of alcohol to exploit vulnerable women [31].

Recent randomized controlled trials demonstrate the effectiveness of staff training programs. The “Safer Bars” intervention in Arizona showed significant reductions in alcohol-related sexual violence through comprehensive bar staff bystander training [32]. Subsequent evaluations have confirmed not only the effectiveness but also the feasibility and acceptability of such training models across different nightlife contexts, showing that bar staff can successfully identify high-risk situations, intervene earlier, and reduce escalation of harassment [29,30]. Moreover, a recent scoping review synthesizing international prevention efforts highlights that staff-focused interventions are most impactful when embedded in broader structural or policy approaches that reshape venue norms and accountability frameworks [32,33,34].

Understanding the epidemiology and social context of party-related sexual violence in Poland is crucial for designing effective prevention strategies and ensuring the right to safety and autonomy.

## 2. Materials and Methods

### 2.1. Study Design and Setting

A cross-sectional survey was carried out in Poland from 18 to 27 March 2025 using the computer-assisted web interview (CAWI) method. Data were collected with a structured questionnaire created by the authors and administered online to a nationwide adult sample. To maintain methodological rigor and safeguard data quality, fieldwork was outsourced to a professional public opinion research company operating a secure online platform. Personalized email invitations were issued, and reminder messages were sent to enhance response. Completion of all items was mandatory, so the dataset contained no missing values.

### 2.2. Participants and Sampling

The study included 1000 adults residing in Poland. Respondents were drawn from a validated online research panel with more than 100,000 registered members. Stratified quota sampling was applied to mirror the national distribution by gender, age, and place of residence as reported by Statistics Poland [12]. When an initially invited person declined, another panelist meeting the same stratification constraints was contacted. Participation was voluntary and anonymous. The approach aligns with prior nationwide cross-sectional surveys in Poland, supporting population-level inference [14,15,16].

### 2.3. Ethical Considerations

The protocol received approval from the Ethics Committee of the Medical University of Warsaw (AKBE/79/2025, 17 March 2025) and the study adhered to the principles of the Declaration of Helsinki.

### 2.4. Questionnaire Development and Measures

The questionnaire was developed following a review of international literature on sexual violence in party-related social settings and adapted to the Polish sociocultural context. Beyond linguistic changes, cultural adaptation involved a forward translation by a bilingual public health researcher, expert review by a multidisciplinary panel (psychiatrists, public health experts, and practitioners in sexual violence prevention) to ensure conceptual and contextual equivalence, and cognitive debriefing with lay respondents to confirm comprehensibility and ecological validity. Similar cultural adaptations were made in other studies [35,36,37]. To improve ecological validity, questions were formulated in accessible, colloquial language. Respondents were explicitly asked about both personal experiences and witnessing incidents, in order to capture the broader spectrum of exposure to sexual misconduct. The instrument comprised four domains: perceptions of risk, referring to the frequency of concerns about experiencing sexual violence during or after social events; preventive strategies, covering self-reported actions taken to increase personal safety while attending or returning from parties; exposure to misconduct and violence, including types of unwanted sexual behaviors and assaults encountered, either personally or as a witness; and sociodemographic variables such as sex, age, educational attainment, sexual orientation, and place of residence. For analytic clarity, the study focused on both subjective fear of sexual assault and reported encounters (witnessed or experienced) with specific forms of misconduct, including verbal harassment, unwanted touching, coercion to consume substances, surreptitious administration of drugs, and forced sex. Content validity was assessed by a panel of psychiatrists, public health experts, and forensic toxicologists. A pilot test with 11 adults was conducted twice over a five-day interval, and based on feedback, one item was refined to better match respondents’ perceptions and reduce ambiguity.

### 2.5. Statistical Analysis

Analyses were conducted in IBM SPSS Statistics, version 29 (Armonk, NY, USA). Categorical variables were summarized using frequencies and percentages. Bivariate relationships between sociodemographic characteristics and study outcomes were examined with chi-square tests. To identify factors independently associated with each outcome, multivariable logistic regression models were fitted. Adjusted odds ratios (aORs) with 95 percent confidence intervals (95 percent CIs) were reported. Statistical significance was defined as *p* < 0.05.

## 3. Results

The study sample comprised 1000 adults, of whom 58.6% were women. The largest age group was respondents aged 30–39 years (25.6%), while those aged 60 or older accounted for 8.8%. More than half of the participants (55.1%) reported higher education, and the vast majority identified as heterosexual (87.9%). Approximately one quarter resided in rural areas, with the remainder distributed across towns and cities of varying sizes. Regional representation reflected all main administrative areas of the country (Table 1).

Most respondents reported at least occasional concerns about experiencing sexual violence while attending social events: 41.4% felt such fears rarely, 16.6% often, and 4.3% almost always, whereas only 37.7% declared never or almost never. A similar pattern was observed when returning home, with 37.9% reporting rare concerns, 18.0% frequent, and 6.8% constant concerns. To increase their safety, participants most often returned with friends (64.2%), informed friends upon arriving safely (53.8%), or notified relatives about their plans (46.5%). Less common strategies included sharing location (30.9%) or carrying pepper spray (20.7%). Reported experiences of misconduct were highly prevalent: more than half had encountered sexual comments and jokes (54.1%) or unwanted touching (53.8%), while nearly one in ten reported drug-facilitated assaults (10.3–10.7%). These findings illustrate the high burden of both perceived risk and actual exposure to sexual violence in party-related contexts (Table 2).

Fear of sexual violence during and after parties varied significantly across sociodemographic groups. Women expressed much higher concern than men: during parties, 28.7% of women reported frequent or constant fear compared with only 9.9% of men (*p* < 0.001), and after parties, 35.5% vs. 9.7%, respectively (*p* < 0.001). Younger participants were also more likely to report fear: among those aged 18–24, 32.6% reported frequent or constant concerns during parties and 39.8% when returning home, compared with only 6.8% and 5.6% in the 60+ group (*p* < 0.001 for trend). Sexual minorities demonstrated elevated levels of fear compared to heterosexual respondents, both at parties and when going home (*p* < 0.001). Residence also mattered: individuals from rural areas more often reported never experiencing such concerns, whereas those from larger cities reported higher levels of fear, with significant variation between groups (*p* < 0.001). These results confirm that gender, age, sexual orientation, and place of residence are strong predictors of perceived risk (Table 3).

Preventive strategies used during and after parties varied across gender, age, education, sexual orientation, and place of residence. Women more frequently than men reported going home with friends (70.3% vs. 55.6, *p* < 0.001), using ride-tracking apps (23.9% vs. 13.8, *p* < 0.001), informing friends after returning home (64.0% vs. 39.4, *p* < 0.001), and carrying pepper spray (24.1% vs. 15.9, *p* = 0.002). Younger respondents (18–24 years) were particularly likely to adopt multiple strategies, including ride-tracking (28.6%, *p* < 0.001) and location sharing (47.1%, *p* < 0.001). In contrast, older participants relied less often on technological solutions. Preventive behaviors were also more common among bisexual respondents compared with heterosexual participants (e.g., notifying friends after returning home: 78.0% vs. 52.9%, *p* = 0.001). Significant geographic variation was observed: residents of large cities more frequently used paid transport (54.5% in cities >500,000 vs. 37.3% in rural areas, *p* < 0.001) and location sharing (42.4% vs. 26.2%, *p* < 0.001). These findings indicate clear sociodemographic disparities in the adoption of personal safety measures (Table 4).

Reported exposure to different forms of abuse or violence during parties and on the way home showed clear sociodemographic differences. Women were significantly more likely than men to report being followed after leaving a party (25.1% vs. 16.9%, *p* = 0.002), being harassed on the way home (50.3% vs. 38.9%, *p* < 0.001), and being pressured to drink excessive amounts of alcohol (47.4% vs. 37.2%, *p* = 0.001). Age patterns indicated that the youngest respondents (18–24 years) most frequently reported harassment (51.5%), while individuals aged 60+ were least likely to report any form of misconduct (*p* < 0.001 for trend). Respondents with vocational education more often declared being pressured into drug use (28.2%) compared with those with higher education (11.8%, *p* = 0.03). Sexual minorities reported elevated risks: homosexual participants were more likely to report harassment and unwanted pursuit, while bisexual respondents showed the highest levels of being pressured to drink (49.2%) and harassed on the way home (52.5%), with differences significant compared with heterosexual respondents (*p* < 0.05). Geographic variation was less pronounced, although residents of the largest cities reported somewhat higher levels of harassment and unwanted pursuit than rural respondents. Overall, the findings confirm that women, younger participants, and sexual minorities are at significantly higher risk of exposure to sexual misconduct and abuse (Table 5).

Verbal and physical forms of sexual misconduct were frequently reported by respondents. Women were significantly more likely than men to encounter sexual comments and jokes (58.7% vs. 47.6%, *p* < 0.001), persistent harassment despite refusal (53.2% vs. 37.9%, *p* < 0.001), and unwanted touching (63.7% vs. 39.9%, *p* < 0.001). Younger respondents, particularly those aged 18–24, most often reported exposure to all three forms (e.g., unwanted touching: 68.4% vs. 39.8% in the 60+ group, *p* < 0.001). Educational level was not strongly associated with these experiences, though sexual orientation played a role: bisexual participants most frequently reported sexual comments (71.2%), harassment (66.1%), and unwanted touching (74.6%), all significantly higher compared with heterosexual respondents (*p* < 0.01). Geographic patterns showed that respondents from the largest cities reported higher exposure, especially to sexual comments (62.8% vs. 50.4% in rural areas, *p* = 0.03). Reports of indecent exposure and forced sex were less common overall (around 20% and 12%, respectively) and did not differ significantly across most subgroups. These findings highlight the disproportionate burden of sexual harassment and unwanted physical contact on women, youth, and sexual minorities (Table 6).

In bivariable and multivariable logistic regression, several factors were significantly associated with experiencing or witnessing sexual violence during parties. Female gender was a strong predictor, with women having almost twice the odds compared to men (aOR: 1.91; 95% CI: 1.39–2.63; *p* < 0.001). Younger age groups were at the highest risk: respondents aged 18–24 had nearly a threefold higher risk compared with those aged 60+ (aOR: 2.80; 95% CI: 1.53–5.12; *p* < 0.001), and elevated odds were also observed for the 25–29, 30–39, 40–49, and 50–59 age groups (all *p* < 0.05). Educational level and sexual orientation were not significantly associated with exposure after adjusting for covariates. Place of residence showed no consistent association, though a trend toward higher odds was observed in large cities. At the regional level, living in the northern administrative region was linked to nearly a twofold increase in risk (aOR: 1.96; 95% CI: 1.08–3.57; *p* = 0.03) compared with the central region. These findings highlight that women, younger adults, and residents of northern Poland are at disproportionate risk of encountering sexual violence or misconduct in party contexts (Table 7).

## 4. Discussion

This study provides one of the first comprehensive assessments of exposure to sexual misconduct and violence in party-related social settings in Poland. Our prevalence findings align with international research while revealing important context-specific patterns. The overall prevalence of experiencing sexual violence in our Polish sample (study results) compares to 58.0% reported in the study conducted in England [26]. The findings demonstrate that both concerns about sexual victimization and actual experiences of harassment and abuse are widespread. More than half of respondents reported encountering sexual comments, jokes, or unwanted touching, and nearly one in ten had experienced or witnessed drug-facilitated sexual assaults. These results confirm that sexual misconduct is not a marginal problem but rather a structural feature of party environments, consistent with international evidence showing that night-time leisure settings often function as high-risk environments for gender-based and sexual violence [1,2,14,16].

This study provides several original contributions to the existing literature. First, it is the first nationwide study in Poland to assess both subjective fear and direct or witnessed exposure to sexual violence in party-related social settings using a population-based sample rather than a student or venue-based cohort. Second, unlike most prior research conducted in single cities or nightlife districts, our data capture regional variation, showing that exposure is not uniformly distributed but shaped by contextual factors such as local drinking cultures and event infrastructure. Third, this is one of the few European datasets documenting the prevalence of suspected drug-facilitated assaults in a general adult sample, rather than in convenience or emergency-room samples. Together, these findings offer new empirical evidence on the epidemiology of sexual violence in social nightlife-adjacent contexts and help clarify which structural determinants may require policy-level intervention.

Clear sociodemographic patterns emerged in this study. Women consistently reported higher levels of fear, harassment, and unwanted physical contact compared with men, and multivariable analyses confirmed female gender as an independent risk factor for exposure to sexual violence (aOR: 1.91; 95% CI: 1.39–2.63). These findings align with the European Union Agency for Fundamental Rights (FRA) surveys, which show that women experience sexual harassment in public spaces significantly more often [7,37]. Similarly, young adults were disproportionately affected, with those aged 18–24 having almost a threefold increased risk compared with participants over 60. This corresponds with studies and communications indicating that young people are especially vulnerable in party contexts due to higher rates of social participation, alcohol consumption, and engagement in settings where sexualized behavior is more prevalent [8,38,39].

Among sexual minorities, bisexual respondents reported the highest level of violent experiences. This may be explained not only by the generally higher vulnerability of LGBTQ+ populations to violence in public spaces, but also by the issue of “double discrimination”—the increased stigmatization of bisexual individuals in both heterosexual and LGBTQ+ spaces [9,40]. This underlines the need for prevention strategies that explicitly address homophobia and biphobia as additional sources of aggression.

All the aforementioned “at-risk” groups reported more frequent use of preventative behaviors, such as returning home with friends, notifying others of their whereabouts (including technologically enabled strategies such as ride-tracking and location sharing), or carrying pepper spray. However, despite widespread adoption of such behaviors, exposure to harassment and violence remained high, suggesting that individual-level protective strategies are insufficient. This observation is consistent with intervention research demonstrating that structural approaches are more effective in reducing sexual violence.

International evidence strongly supports venue-level and community-based interventions. The STOP-SV (Staff Training on Prevention of Sexual Violence) project, evaluated across three European countries (Czech Republic, Portugal, and Spain), demonstrated significant improvements in nightlife workers’ attitudes and intervention capacity. Quigg et al., (2021) showed that a two-hour training program was associated with decreased acceptance of sexual violence myths (*p* < 0.01; small effect size d = 0.22) and increased readiness and confidence to intervene (*p* < 0.001; medium effect sizes d = −0.46 and d = 0.32, respectively) [40]. Notably, 56.0% of nightlife worker participants reported personal experiences of sexual violence in nightlife settings, underscoring the importance of workplace-targeted prevention. Additional evidence from venue-based interventions also demonstrates promise: Powers and Leili (2018) found that bar staff recognized their potential role in prevention but cited barriers such as lack of training and protocols; their evaluation of a bystander program showed positive impacts on rape myth rejection and intervention willingness [30]. Systematic reviews further support the effectiveness of bystander programs among adolescents and college students, though effects are moderate [41]. Collectively, these findings suggest that community-based, multi-component approaches are more effective than individual strategies alone [11,12,29,30].

In the Polish context, this evidence is particularly relevant given the recent shift toward a consent-based legal definition of rape, which creates favorable conditions for embedding prevention efforts within nightlife infrastructures. International models such as Safer Bars and STOP-SV could be adapted through mandatory bar-staff training focused on early recognition of predatory behavior, venue-level safeguarding protocols, and clear reporting pathways for patrons. Importantly, such measures shift responsibility for safety away from potential victims and toward institutional accountability, aligning prevention efforts in Poland with a structural public health approach.

Approximately 10% of respondents reported experiencing drug-facilitated sexual assault, indicating that this issue is not incidental in Poland and requires urgent attention from both researchers and legislators. Consistent with prior research, alcohol emerged as the most common substance involved in party-related sexual assault [1,13]. In our study, over 40% of respondents reported being pressured to drink excessively, highlighting alcohol’s central role in facilitating harassment and abuse. Regional variation was also observed: living in the northern region of Poland was associated with nearly double the odds of exposure to sexual violence compared with central regions (aOR: 1.96; 95% CI: 1.08–3.57). Such heterogeneity may reflect differences in drinking cultures, party infrastructure, or preventive policies.

Taken together, the results demonstrate high rates of both perceived danger and actual exposure to sexual violence in party-related social settings, alongside the limited effectiveness of individual precautionary measures. This underscores the need for systemic interventions aimed at improving safety. Evidence from other countries indicates that structural and cultural interventions, such as alcohol policy reforms, venue-based staff training, or bystander empowerment campaigns, can effectively reduce incidents of harassment and violence [11,30]. Moreover, strategies that place responsibility for safety primarily on potential victims are not only less effective but also perpetuate narratives of victim-blaming, rather than focusing accountability on perpetrators.

Finally, sexual harassment and violence are consistently associated with adverse mental health outcomes, including post-traumatic stress disorder, depression, anxiety, and substance use disorders. The clear presence of at-risk groups in our study reinforces the interpretation of party-related sexual violence not only as a public health issue but also as a human rights concern. Our findings indicate that party-related sexual violence in Poland reflects both local dynamics and broader global patterns.

## 5. Limitations

Several limitations should be considered when interpreting the findings of this study. First, the cross-sectional design precludes causal inferences regarding the observed associations. Second, data were collected using self-reported measures, which may be influenced by recall or social desirability bias, particularly given the sensitive nature of sexual violence. Third, although quota-based sampling ensured alignment with national benchmarks for age, gender and region, some groups may still be relatively underrepresented, particularly individuals with lower educational attainment and non-heterosexual orientations. This has implications for prevalence estimates: sexual violence may be underestimated among individuals with lower educational attainment due to greater barriers to disclosure, whereas smaller subsamples of sexual minority respondents may produce less stable estimates with potential fluctuation in either direction depending on disclosure norms. Moreover, the exclusive use of an online research panel entails a risk of digital exclusion bias, as individuals with limited or unstable internet access are less likely to participate in CAWI surveys. Because digital exclusion overlaps with socioeconomic disadvantage and geographic peripherality, the true prevalence of sexual victimization in party-related social settings may be higher than reported. Fourth, the survey focused on selected forms of misconduct and may not have captured the full spectrum of experiences related to sexual violence in party-related social contexts. The issue of cross-cultural comparability arises not only from linguistic differences but also from variation in social norms surrounding bodily autonomy, thresholds for defining harassment, and the regulatory and architectural characteristics of nightlife and social venues across countries. As a result, prevalence rates obtained with culturally adapted tools may not be directly comparable with those from jurisdictions differing in alcohol-serving regulations, bystander norms, or venue-level safeguarding policies. Future research could address this limitation by employing harmonized multi-country questionnaire modules or parallel administration of both locally adapted and internationally standardized instruments to assess measurement equivalence.

## 6. Conclusions

This study demonstrates that concerns about sexual victimization and actual exposure to sexual misconduct are common among adults participating in party activities in Poland. Women, younger adults, and sexual minorities bear a disproportionate burden of risk, while regional variation suggests that contextual factors may further shape vulnerability. Despite the widespread use of preventive strategies, including traveling with friends, notifying others, and using mobile technologies, the prevalence of harassment and violence remains high, indicating that these individual measures are insufficient to ensure safety.

These findings highlight the urgent need for multilevel interventions that go beyond individual precautionary behaviors. Preventive strategies should involve structural changes in party venues, bystander intervention programs, and broader public health and policy responses aimed at reducing social tolerance for harassment and sexual violence. In light of the recent legal shift toward a consent-based definition of rape, there is now a critical opportunity for coordinated policy action to embed these changes at the systemic level. Special attention should be given to protecting groups at elevated risk, particularly women, young people, and sexual minorities.

## Figures and Tables

**Table 1:** Sociodemographic characteristics of the study population (*n* = 1000).

	***n***	**%**
**Gender**		
Female	586	58.6%
Male	414	41.4%
**Age (in years)**		
18–24	206	20.6
25–29	140	14.0
30–39	256	25.6
40–49	192	19.2
50–59	118	11.8
60+	88	8.8
**Educational level**		
primary	43	4.3
vocational	39	3.9
secondary	367	36.7
higher	551	55.1
**Self-declared sexual orientation**		
heterosexuality	879	87.9
homosexuality	37	3.7
bisexuality	59	5.9
other	13	1.3
refusal to respond	12	1.2
**Place of residence**		
rural area	260	26.0
city with up to 20,000 residents	102	10.2
city from 20,000 to 100,000 residents	188	18.8
city from 100,000 to 500,000 residents	219	21.9
city with over 500,000 residents	231	23.1
**Administrative region of the country**		
central	256	25.6
south	208	20.8
east	172	17.2
north-west	138	13.8
south-west	104	10.4
north	122	12.2

**Table 2:** Concerns about sexual violence, preventive behaviors, and reported exposure to sexual misconduct during or after social events (*n* = 1000).

	**% (** ***n*** **)**
**How Often, While at a Party, Do You Worry That You Might Experience Sexual Violence?**	
Never or almost never	37.7% (377)
Rarely—sometimes, but not often	41.4% (414)
Often—more often than not	16.6% (166)
Always or almost always	4.3% (43)
**How often, when going home from a party, do you worry that you might experience sexual violence?**	
Never or almost never	37.3% (373)
Rarely—sometimes, but not often	37.9% (379)
Often—more often than not	18.0% (180)
Always or almost always	6.8% (68)
**What do you do during or after parties to stay safe? (multiple choice)**	
Go home with friends	64.2% (642)
Take a taxi/Uber/Bolt/other paid transport	43.2% (432)
Use the ride-tracking option in the app	19.7% (197)
Tell friends when you arrive home safely	53.8% (538)
Tell relatives about the party and return time	46.5% (465)
Carry pepper spray	20.7% (207)
Share your location with someone you trust	30.9% (309)
Other	1.9% (19)
None of the above	6.6% (66)
**What forms of abuse or violence have you seen or experienced at parties or on the way home? (‘Seen’ means as a witness or victim)**	
Taking intimate photos without consent/secretly	18.1% (181)
Being followed after leaving a party	21.7% (217)
Being harassed on the way home	45.6% (456)
Secretly being given drugs (e.g., in drinks) leading to sexual activity	10.7% (107)
Secretly being given a “date rape drug” leading to sexual activity	10.3% (103)
Being pressured to drink too much alcohol	43.2% (432)
Being pressured to take too many drugs	13.1% (131)
Being taken advantage of while intoxicated	18.3% (183)
Sexual comments and “jokes”	54.1% (541)
Persistent harassment despite refusal	46.9% (469)
Unwanted touching (e.g., groping, grabbing buttocks)	53.8% (538)
Unwanted exposure	20.9% (209)
Being forced to have sex	12.3% (123)

**Table 3:** Demographic profile of respondents completing items on sexual violence, preventive behaviors, and reported exposure to sexual misconduct during or after social events (*n* = 1000).

	How Often, While at a Party, Do You Worry That You Might Experience Some Form of Sexual Violence?					How Often, When Going Home From a Party, Do You Worry That You Might Experience Some Form of Sexual Violence?				
	Never or Almost Never	Rarely—From Time to Time, but Less Often Than Not	Often—Not Always, but More Often Than Not	Always or Almost Always	*p*	Never or Almost Never	Rarely—From Time to Time, but Less Often Than Not	Often—Not Always, but More Often Than Not	Always or Almost Always	*p*
**gender**										
female (*n* = 586)	22.2	49.1	22.0	6.7	**<0.001**	19.8	44.7	24.7	10.8	**<0.001**
male (*n* = 414)	59.7	30.4	8.9	1.0		62.1	28.3	8.5	1.2	
**age**										
18–24 (*n* = 206)	23.3	44.2	24.8	7.8	**<0.001**	22.3	37.9	24.8	15.0	**<0.001**
25–29 (*n* = 140)	31.4	42.9	22.9	2.9		29.3	39.3	22.1	9.3	
30–39 (*n* = 256)	36.7	44.1	15.2	3.9		35.9	41.8	18.4	3.9	
40–49 (*n* = 192)	34.9	46.9	13.0	5.2		41.7	35.4	16.7	6.3	
50–59 (*n* = 118)	50.0	36.4	11.9	1.7		45.8	40.7	12.7	0.8	
60+ (*n* = 88)	73.9	19.3	5.7	1.1		68.2	26.1	4.5	1.1	
**Educational level**										
primary (*n* = 43)	30.2	34.9	23.3	11.6	0.1	25.6	32.6	27.9	14.0	**0.3**
vocational (*n* = 39)	53.8	30.8	11.03	5.1		48.7	30.8	12.8	7.7	
secondary (*n* = 367)	37.1	40.6	18.5	3.8		38.4	37.6	17.7	6.3	
higher (*n* = 551)	37.6	43.2	15.2	4.0		36.7	39.0	17.8	6.5	
**Self-declared sexual orientation**										
heterosexuality (*n* = 879)	39.2	41.8	14.9	4.1	**<0.001**	39.5	38.0	16.7	5.8	**<0.001**
homosexuality (*n* = 37)	32.4	24.3	40.5	2.7		21.6	40.5	27.0	10.8	
bisexuality (*n* = 59)	15.3	54.2	27.1	3.4		13.6	37.3	33.9	15.3	
other (*n* = 13)	30.8	30.8	23.1	15.4		23.1	46.2	15.4	15.4	
refusal to respond (*n* = 12)	58.3	16.7	8.3	16.7		58.3	16.7	8.3	16.7	
**Place of residence**										
rural area (*n* = 260)	46.5	36.9	14.2	2.3	**0.07**	47.7	38.1	10.0	4.2	**<0.001**
city up to 20,000 residents (*n* = 102)	36.3	42.2	16.7	4.9		39.2	32.4	21.6	6.9	
city from 20,000 to 100,000 residents (*n* = 188)	34.6	45.7	16.0	3.7		31.9	40.4	20.7	6.9	
city from 100,000 to 500,000 residents (*n* = 219)	38.8	40.2	15.5	5.5		40.2	34.7	17.8	7.3	
city with over 500,000 residents (*n* = 231)	29.9	43.7	20.8	5.6		26.4	41.1	23.4	9.1	
**Administrative region of the country**										
central (*n* = 256)	37.1	43.0	16.0	3.9	0.8	35.9	36.7	22.7	4.7	**0.2**
south (*n* = 208)	36.5	41.3	17.8	4.3		36.1	38.0	19.7	6.3	
east (*n* = 172)	40.1	41.9	15.1	2.9		45.3	37.8	10.5	6.4	
north-west (*n* = 138)	38.4	42.0	12.3	7.2		39.1	35.5	16.7	8.7	
south-west (*n* = 104)	35.6	38.5	23.1	2.9		29.8	40.4	20.2	9.6	
north (*n* = 122)	38.5	39.3	17.2	4.9		35.2	41.0	15.6	8.2	

**Table 4:** Preventive behaviors during and after parties, stratified by sociodemographic characteristics (*n* = 1000).

What Do You Do During or After Parties to Increase Your Safety? (Multiple Choice Question)																
	Go Home from the Party with Friends		Go Home from the Party by Taxi		Use the Ride-Tracking Option in the App		Inform Friends That You Arrived Home Safely		Tell Relatives That You Are Going to a Party and When You Plan to Return		Carry Pepper Spray		Share Your Location with a Trusted Person via Phone		None of the Listed Methods	
**gender**																
female (*n* = 586)	70.3	**<0.001**	45.7	0.05	23.9	**<0.001**	64.0	**<0.001**	53.6	**<0.001**	24.1	**0.002**	39.8	**<0.001**	4.8	**0.006**
male (*n* = 414)	55.6		39.6		13.8		39.4		36.5		15.9		18.4		9.2	
**age**																
18–24 (*n* = 206)	69.4	**0.02**	41.7	**0.03**	28.6	**<0.001**	65.0	**<0.001**	55.8	**0.02**	28.6	**<0.001**	47.1	**<0.001**	6.3	0.6
25–29 (*n* = 140)	55.0		46.4		28.6		60.7		43.6		29.3		42.1		6.4	
30–39 (*n* = 256)	60.5		45.7		19.5		51.2		47.7		20.7		29.3		4.7	
40–49 (*n* = 192)	63.5		44.8		14.6		50.5		44.8		15.1		22.9		10.2	
50–59 (*n* = 118)	66.9		46.6		12.7		45.8		35.6		16.1		20.3		6.8	
60+ (*n* = 88)	75.0		26.1		5.7		42.0		44.3		6.8		11.4			
**Educational level**																
primary (*n* = 43)	72.1	0.2	41.9	**0.01**	16.3	0.3	51.2	0.2	55.8	0.1	14.0	0.6	37.2	0.08	7.0	0.9
vocational (*n* = 39)	74.4		28.2		10.3		38.5		30.8		15.4		12.8		7.7	
secondary (*n* = 367)	66.2		38.7		18.8		52.9		47.4		21.0		31.1		6.8	
higher (*n* = 551)	61.5		47.4		21.2		55.7		46.3		21.4		31.6		6.4	
**Self declared sexual orientation**																
heterosexuality (*n* = 879)	65.1	0.06	43.3	0.3	18.8	0.2	52.9	**0.001**	46.1	0.4	20.8	0.5	29.6	**0.02**	2.2	0.6
homosexuality (*n* = 37)	45.9		45.9		21.6		40.5		45.9		13.5		35.1		0.0	
bisexuality (*n* = 59)	69.5		47.5		27.1		78.0		57.6		23.7		45.8		0.0	
other (*n* = 13)	46.2		30.8		38.5		38.5		38.5		30.8		53.8		0.0	
refusal to respond (*n* = 12)	50.0		16.7		25.0		58.3		33.3		8.3		16.7		0.0	
**Place of residence**																
rural area (*n* = 260)	65.4	0.6	37.3	**<0.001**	18.1	**0.007**	47.7	**0.02**	40.4	0.05	18.1	0.5	26.2	**<0.001**	7.3	0.9
city up to 20,000 residents (*n* = 102)	65.7		30.4		18.6		50.0		52.0		22.5		28.4		6.9	
city from 20,000 to 100,000 residents (*n* = 188)	66.0		35.1		15.4		52.7		43.1		21.8		26.6		5.9	
city from 100,000 to 500,000 residents (*n* = 219)	65.3		51.1		16.9		54.8		47.9		18.7		29.2		5.9	
city with over 500,000 residents (*n* = 231)	59.7		54.5		28.1		62.3		52.4		23.8		42.4		6.9	
**Administrative region of the country**																
central (*n* = 256)	61.7	0.4	38.7	**0.02**	24.2	0.1	50.4	**0.04**	43.8	0.7	18.0	0.4	33.6	0.3	9.4	0.2
south (*n* = 208)	69.2		47.1		18.8		59.6		49.0		22.1		32.7		3.4	
east (*n* = 172)	61.6		46.5		17.4		45.4		43.6		22.7		30.2		7.6	
north-west (*n* = 138)	65.9		52.2		23.2		56.5		47.1		23.2		32.6		5.8	
south-west (*n* = 104)	58.7		39.4		16.3		60.6		51.9		24.0		29.8		5.8	
north (*n* = 122)	64.2		34.4		13.9		54.1		46.7		15.6		22.1		6.6	

**Table 5:** Reported exposure to sexual misconduct and violence during and after parties, stratified by sociodemographic characteristics (*n* = 1000).

What Forms of Abuse or Violence Have You Encountered at Parties and/or When Returning from Them? By “Encountered” We Mean Situations Where You Were a Witness or Personally Experienced Such Behaviors.																
	Recording or Taking Intimate Photos Without Consent/Secretly		Being Followed After Leaving a Party		Being Harassed on the Way Home		Being Secretly Given Intoxicating DRUGS (e.g., in Drinks) Leading to Sexual Intercourse in Any Form		Being Secretly Given a So-Called “Date Rape Drug” Leading to Sexual Intercourse in Any Form		Being Pressured to Drink Excessive Amounts of ALCOHOL		Being Pressured to Use Excessive Amounts of Drugs		Exploitation of a Person Under the Influence	
**gender**																
female (*n* = 586)	18.1	0.9	25.1	**0.002**	50.3	**<0.001**	11.3	0.5	10.4	0.9	47.4	**0.001**	12.5	0.5	18.9	0.5
male (*n* = 414)	18.1		16.9		38.9		9.9		10.1		37.2		14.0		17.4	
**age**																
18–24 (*n* = 206)	23.3	0.09	24.8	0.3	51.5	0.8	9.7	0.3	7.3	**0.02**	49.5	0.3	15.5	0.2	20.9	0.8
25–29 (*n* = 140)	16.4		20.7		48.6		12.1		12.9		40.7		15.0		20.0	
30–39 (*n* = 256)	18.4		24.2		45.3		13.3		10.9		44.1		12.9		18.0	
40–49 (*n* = 192)	19.3		21.9		45.8		11.5		15.6		40.1		14.1		17.2	
50–59 (*n* = 118)	15.3		17.8		41.5		6.8		5.9		43.2		11.9		15.3	
60+ (*n* = 88)	9.1		13.6		33.0		6.8		5.7		36.4		4.5		17.0	
**Educational level**																
primary (*n* = 43)	25.6	0.2	25.6	0.9	39.5	0.6	7.0	0.4	14.0	0.6	44.2	0.5	9.3	**0.03**	11.6	0.5
vocational (*n* = 39)	28.2		23.1		38.5		15.4		10.3		51.3		28.2		23.1	
secondary (*n* = 367)	18.3		20.7		45.0		12.3		11.4		40.6		13.9		19.3	
higher (*n* = 551)	16.7		22.0		47.0		9.6		9.3		44.3		11.8		17.8	
**Self declared sexual orientation**																
heterosexuality (*n* = 879)	18.3	0.07	21.2	0.2	45.5	**0.02**	11.3	0.5	10.8	0.5	42.5	**0.003**	13.0	0.07	18.4	0.4
homosexuality (*n* = 37)	27.0		35.1		51.4		8.1		10.8		45.9		21.6		21.6	
bisexuality (*n* = 59)	10.2		23.7		52.5		6.8		3.4		49.2		8.5		20.3	
other (*n* = 13)	30.8		23.1		46.2		7.7		7.7		84.6		30.8		7.7	
refusal to respond (*n* = 12)	0.0		8.3		0.0		0.0		8.3		8.3		0.0		0.0	
**Place of residence**																
rural area (*n* = 260)	18.1	0.8	18.1	0.1	46.2	0.7	11.9	0.9	10.4	0.9	45.0	0.6	12.3	0.7	16.9	0.6
city up to 20,000 residents (*n* = 102)	14.7		22.5		41.2		8.8		7.8		44.1		16.7		17.6	
city from 20,000 to 100,000 residents (*n* = 188)	19.1		17.6		44.7		10.6		11.7		38.3		12.8		19.7	
city from 100,000 to 500,000 residents (*n* = 219)	16.9		25.1		44.3		9.6		11.0		42.0		11.4		16.0	
city with over 500,000 residents (*n* = 231)	19.9		25.5		48.9		11.3		9.5		45.9		14.3		21.2	
**Administrative region of the country**																
central (*n* = 256)	16.0	0.9	19.9	0.8	42.6	0.9	10.5	0.8	7.8	0.4	43.0	0.9	12.9	0.6	17.2	0.5
south (*n* = 208)	18.8		22.6		46.2		10.6		10.6		47.1		11.1		21.2	
east (*n* = 172)	19.2		21.5		47.7		11.0		9.3		41.3		16.9		15.7	
north-west (*n* = 138)	18.1		26.1		45.7		8.0		14.5		42.0		13.8		20.3	
south-west (*n* = 104)	19.2		20.2		44.2		10.6		10.6		43.3		10.6		21.2	
north (*n* = 122)	18.9		20.5		49.2		13.9		11.5		41.0		13.1		14.8	

**Table 6:** Reported exposure to verbal and physical forms of sexual misconduct during and after parties, stratified by sociodemographic characteristics (*n* = 1000).

What Forms of Abuse or Violence Have You Encountered at Parties and/or When Returning from Them? By “Encountered” We Mean Situations Where You Were a Witness or Personally Experienced Such Behaviors.										
	Sexual Comments and “Jokes”		Persistent Harassment Despite Refusal		Unwanted Touching (e.g., Groping, GRABBING Buttocks)		Unwanted Exposure		Being Forced to Have Sex	
**gender**										
female (*n* = 586)	58.7	**<0.001**	53.2	**<0.001**	63.7	**<0.001**	22.2	0.2	12.3	0.9
male (*n* = 414)	47.6		37.9		39.9		19.1		12.3	
**age**										
18–24 (*n* = 206)	67.0	**<0.001**	59.2	**<0.001**	68.4	**<0.001**	23.8	0.2	13.1	0.7
25–29 (*n* = 140)	57.9		47.9		56.4		21.4		15.7	
30–39 (*n* = 256)	49.2		48.0		52.0		21.1		10.9	
40–49 (*n* = 192)	50.5		44.8		46.9		22.4		12.0	
50–59 (*n* = 118)	54.2		39.0		50.8		20.3		9.3	
60+ (*n* = 88)	39.8		28.4		39.8		10.2		13.6	
**Educational level**										
primary (*n* = 43)	55.8	0.9	51.2	0.8	58.1	0.9	32.6	0.3	14.0	0.7
vocational (*n* = 39)	48.7		41.0		51.3		23.1		17.9	
secondary (*n* = 367)	54.0		47.4		52.6		21.0		11.7	
higher (*n* = 551)	54.4		46.6		54.4		19.8		12.2	
**Self declared sexual orientation**										
heterosexuality (*n* = 879)	52.6	**0.01**	46.2	**<0.001**	52.3	**0.001**	21.0	0.5	12.3	0.7
homosexuality (*n* = 37)	64.9		48.6		56.8		24.3		13.5	
bisexuality (*n* = 59)	71.2		66.1		74.6		20.3		15.3	
other (*n* = 13)	69.2		46.2		76.9		23.1		7.7	
refusal to respond (*n* = 12)	33.3		0.0		25.0		0.0		0.0	
**Place of residence**										
rural area (*n* = 260)	50.4	**0.03**	44.2	0.1	51.2	0.2	16.5	0.2	13.5	0.9
city up to 20,000 residents (*n* = 102)	52.9		57.8		53.9		22.5		11.8	
city from 20,000 to 100,000 residents (*n* = 188)	54.8		46.3		54.8		25.5		12.2	
city from 100,000 to 500,000 residents (*n* = 219)	49.3		42.9		49.3		21.0		11.0	
city with over 500,000 residents (*n* = 231)	62.8		49.4		60.2		21.2		12.6	
**Administrative region of the country**										
central (*n* = 256)	54.7	0.9	48.0	0.9	52.7	0.4	19.9	0.7	14.1	0.5
south (*n* = 208)	54.8		45.2		58.7		23.1		11.5	
east (*n* = 172)	52.9		47.1		48.3		20.9		10.5	
north-west (*n* = 138)	52.2		44.9		51.4		18.8		10.1	
south-west (*n* = 104)	52.9		47.1		58.7		17.3		10.6	
north (*n* = 122)	56.6		49.2		54.1		24.6		16.4	

**Table 7:** Factors associated with experiencing or witnessing sexual violence or misconduct during parties (*n* = 1000).

	Encountering (Experiencing or Witnessing) Some Form of Violence or Sexual Abuse While Partying					
			Bivariable Logistic Regression		Multivariable Logistic Regression	
	%	%	OR (95%CI)	*p*	aOR (95%CI)	*p*
**Gender**						
female (*n* = 586)	84.3	**<0.001**	2.09 (1.53–2.85)	**<0.001**	1.91 (1.39–2.63)	**<0.001**
male (*n* = 414)	72.0		Reference		Reference	
**Age [years]**						
18–24 (*n* = 206)	85.9	**<0.001**	3.49 (1.94–6.26)	**<0.001**	2.80 (1.53–5.12)	**<0.001**
25–29 (*n* = 140)	82.9		2.76 (1.49–5.12)	**0.001**	2.50 (1.33–4.70)	**0.004**
30–39 (*n* = 256)	77.3		1.95 (1.16–3.29)	**0.01**	1.77 (1.04–3.02)	**0.04**
40–49 (*n* = 192)	78.1		2.04 (1.17–3.55)	**0.01**	1.90 (1.08–3.33)	**0.03**
50–59 (*n* = 118)	80.5		2.36 (1.26–4.43)	**0.007**	2.38 (1.26–4.52)	**0.01**
60+ (*n* = 88)	63.6		Reference		Reference	
**Educational level**						
primary (*n* = 43)	83.7	0.7	1.31 (0.57–3.03)	0.5		
vocational (*n* = 39)	82.1		1.17 (0.50–2.71)	0.7		
secondary (*n* = 367)	77.7		0.89 (0.64–1.22)	0.5		
higher (*n* = 551)	79.7		Reference			
**Self-declared sexual orientation**						
heterosexuality (*n* = 879)	78.7	0.3	0.78 (0.47–1.28)	0.3		
other (*n* = 121)	82.6		Reference			
**Place of residence**						
rural area (*n* = 260)	76.9	0.3	0.70 (0.45–1.09)	0.1		
city up to 20,000 residents (*n* = 102)	84.3		1.13 (0.60–2.12)	0.7		
city from 20,000 to 100,000 residents (*n* = 188)	77.7		0.73 (0.45–1.18)	0.2		
city from 100,000 to 500,000 residents (*n* = 219)	77.2		0.71 (0.45–1.13)	0.1		
city with over 500,000 residents (*n* = 231)	82.7		Reference			
**Administrative region of the country**						
central (*n* = 256)	76.6	0.1	Reference		Reference	
south (*n* = 208)	82.7		1.46 (0.92–2.32)	0.1	1.42 (0.90–2.28)	0.1
east (*n* = 172)	76.2		0.98 (0.62–1.54)	0.9	1.05 (0.66–1.68)	0.8
north-west (*n* = 138)	75.4		0.94 (0.58–1.52)	0.8	1.00 (0.61–1.63)	0.9
south-west (*n* = 104)	80.8		1.29 (0.73–2.27)	0.4	1.31 (0.73–2.33)	0.4
north (*n* = 122)	86.1		1.89 (1.05–3.41)	**0.03**	1.96 (1.08–3.57)	**0.03**

## Data Availability

The data presented in this study are available on request from the corresponding author. The original dataset cannot be made publicly available due to binding ethical and legal constraints arising from the informed consent procedure and national data protection regulations.
